# Intraocular Pressure and Refractive Changes Following Orbital Decompression with Intraconal Fat Excision

**DOI:** 10.2174/1874364100802010073

**Published:** 2008-04-16

**Authors:** Suresh Sagili, Jean-Louis DeSousa, Raman Malhotra

**Affiliations:** Queen Victoria Hospital, East Grinstead, West Sussex, UK

**Keywords:** Orbital decompression, intraconal fat excision, intraocular pressure, refraction, proptosis.

## Abstract

The purpose of this study was to measure the changes in intraocular pressure (IOP) and refraction following orbital decompression for thyroid orbitopathy.

**Methods:**

Retrospective review of 18 eyes in 10 consecutive patients who underwent orbital decompression including intraconal fat excision for proptosis secondary to thyroid orbitopathy. IOP using tonopen, exophthalmometry, autorefraction and autokeratometry measurements were performed at 1-week, 1-month and 3-months after surgery.

**Results:**

There was no statistically significant difference between the preoperative and postoperative IOP at 3 months. There were no significant differences found between preoperative and post operative keratometry readings or automated refraction following orbital decompression.

**Conclusion:**

Our study did not find a significant change in IOP and refraction following orbital decompression with intraconal fat excision. A larger prospective study is required in order to evaluate the role of intraconal fat excision in reducing IOP due to it’s potential role in patients with concurrent glaucoma.

## INTRODUCTION

Thyroid orbitopathy (TO) may manifest as soft tissue or eyelid changes, myopathy, ocular surface changes, raised intraocular pressure (IOP) and optic neuropathy. IOP rise may be due to increased episcleral venous pressure [1], indentation by hypertrophied extraocular muscle [2,3], trabecular meshwork changes [4] and genetic predisposition [4]. Furthermore, hypermetropic shift has been described in TO, related to flattening of the posterior pole by enlarged muscle and intraorbital fat volume [5]. Robert *et al*. [6] have reported a 17.6% drop in IOP following orbital fat resection in TO. Refractive changes following orbital decompression have also been reported [7,8]. The purpose of this study was to report our experience in IOP and refraction following orbital decompression with intraconal fat excision for TO.

## MATERIALS AND METHODOLOGY

We carried out a retrospective review of 18 eyes of 10 consecutive patients undergoing orbital decompression for stable proptosis secondary to TO during a 24 month period. Surgery was performed by a single surgeon (RM). Intraconal orbital fat excision was performed *via *a conjunctival incision in the inferior fornix or a swinging eyelid approach. The inferotemporal intraconal space was opened, presenting orbital fat excised aided by monopolar cautery, prior to any planned bone decompression. The volume of intraconal fat excised was accurately recorded. Medial orbital wall decompression was performed *via *a transcaruncular incision and lateral wall burring through an upper eyelid skin crease or swinging eyelid approach. If a three-wall decompression was planned, the orbital floor was removed *via *a swinging eyelid flap, preserving the inferomedial strut.

IOP (using TONO-PEN^®^), Hertel exophthalmometry, autorefraction (Nidek) and autokeratometry (Nidek) measurements were performed preoperatively and at 1 week, 1 month and 3 months after surgery. Intraocular pressure was measured with a TONO-PEN^®^ applanation tonometer to ensure central corneal measurements in the primary gaze in all cases. These measurements were performed by the same individual on the same machine at approximately the same time of the day at every visit.

## STATISTICAL ANALYSIS

Comparison between the preoperative and postoperative IOP data was performed using a Student’s t test. All tests were 2 tailed. Comparison of refractive data was performed using Wilcoxon signed rank test. A level of P<0.05 was accepted as statistically significant. In cases of unilateral surgery, IOP changes in the contra lateral unoperated eye were also analysed in order to exclude any postoperative change in the absence of the decompression procedure.

## RESULTS

There were 9 females and 1 male with a mean age of 49 years (range 32 to 70years). Intraconal fat excision was carried out in all cases. In addition seven eyes (4 patients) underwent two-wall (medial and lateral) balanced bone removal decompression and 5 eyes (4 patients) underwent three-wall decompression. Six eyes (3 patients) underwent intraconal fat excision only. Mean orbital fat excised was 1.7±0.6ml (range 0.5ml to 3ml). Table **1** shows the relation between the decompression procedures, amount of fat resected and change in IOP. Two patients had bilateral simultaneous surgery, two patients had unilateral surgery only and 6 patients had bilateral sequential orbital decompression. One patient developed new-onset diplopia, postoperatively.

## REDUCTION IN PROPTOSIS

There was a significant reduction in mean proptosis from 23.4± 2.6mm preoperatively to 19.7± 1.2mm at 3 months postoperatively (p<0.001). The fat excision only group demonstrated a mean reduction in proptosis of 2± 0.8mm, two-wall bone + fat excision group achieved 4± 1.1mm reduction and three wall bone + fat excision group achieved 5± 2.6mm reduction in proptosis at 3 months follow up.

## IOP DATA

The preoperative IOP was less than 21mm in all our patients with an average of 17.6±2.8mmHg. Mean postoperative IOP at 1 week was 16.9± 3.0mmHg and the mean postoperative IOP at 3 months was 16.3±2.9mmHg. There was no statistically significant change in IOP between baseline and 1 week (p = 0.2) and 3months (p = 0.3) postoperatively, respectively. A reduction in IOP was observed in 8 eyes, no change in IOP was found in 6 eyes and an increase in IOP from baseline was observed in 4 eyes at 3 months, postoperatively. The increase in IOP recorded in 4 eyes postoperatively was not clinically significant (1 mmHg increase in IOP recorded in 3 eyes and 2 mmHg rise recorded in 1 eye).

The mean IOP in the contra lateral unoperated eye was 15.3± 3.3mmHg and there was no change at 1 week (15.9±2.9mmHg, p=0.1) postoperatively. There was no significant difference in IOP between the operated and the contra lateral unoperated eye at 1week (p=0.1) postoperatively.

## REFRACTIVE DATA

The majority of eyes (72%, 13 eyes) were myopic (Spherical equivalent, range from -3.00 D to -0.25 D) and did not show a significant change following decompression. There were no significant differences found between preoperative and post operative keratometry readings (K1, p= 0.20), (K2, p = 0.06), keratometry axis (p = 0.45), automated refraction (A1, p=0.89), (A2, p=0.76) or the axis of automated refraction (p=0.42) at 3 months follow up (Table **2**).

## DISCUSSION

In this study we have evaluated the changes in IOP and refraction following orbital decompression. Unlike the previous studies we have used Tonopen to measure the IOP as it is technically easier to ensure that central corneal IOP is recorded in up and down gaze in addition to primary gaze and is sufficiently consistent and accurate when compared to the Goldmann tonometer [9-11]. As our study group included patients undergoing intraconal fat excision only as well as two and three wall bone decompression, we have also studied the IOP change in these subgroups individually. All our patients had a preoperative IOP of less than 21mmHg and did not demonstrate a significant reduction in IOP following orbital decompression. There may therefore be a bias in this cohort towards less of a change in IOP following surgery where pre-operative IOP is not significantly raised, in comparison to congestive cases with “tight orbits”. We used the fellow eye as a control to compare the changes in IOP at 1 week following orbital decompression, but could not find a significant difference in IOP between the operated and the unoperated eyes. The mean amount of orbital fat resected by Robert *et al*. [6] was 6.4±4.5ml whereas we had resected 1.7±0.6ml of orbital fat. The low volume of fat resected in our study group could also be an explanation for the insignificant change in IOP. 72% (13eyes) of our patients had myopic refraction at presentation and did not show a significant change in the refractive state after orbital decompression. There was no significant change in keratometry following orbital decompression.

Several mechanisms have been described for the raised intraocular pressure in thyroid eye disease and there are several reports in literature describing a reduction in IOP following orbital decompression [1,6,12-14]. Danesh-Meyer *et al. *[11] have reported a 6.9% drop in IOP following bone removal orbital decompression in 116 eyes with a preoperative IOP of less than 21mmHg and an even greater reduction in patients with elevated pre operative IOP. Dev *et al.* [13] have reported a mean reduction IOP of 3 mmHg in 22 eyes. The above two were retrospective studies. Robert *et al. *[6] have reported a 16% drop in IOP following fat removal orbital decompression in 42 eyes with a preoperative IOP of less than 21mmHg. This was a prospective study and did not show any correlation between the preoperative proptosis, amount of orbital fat excised and the reduction in IOP. The above studies have used applanation tonometry and there was not enough information provided on the timing of IOP measurement, IOP in downgaze measurements and central corneal thickness. The fellow eye was not used as a control in these studies. The importance of IOP measurement in primary, up and downgaze was stressed by Kalmann *et al.* [1] who demonstrated a reduction in IOP following orbital decompression and inferior rectus recession. As demonstrated by Reader [15], when looking at the slitlamp applanation tonometer, the eyes are not in the primary position but are in mild upgaze during this measurement and could lead to erroneous high pressure recording in so-called “primary position”.

There are very few reports describing refractive changes in TO. Mombaerts *et al. *[7] have reported with-the-rule corneal astigmatism in Graves’ disease possibly caused by soft-tissue fibrosis in the superolateral orbital region which was not influenced by orbital surgery. 56% of our patients had with-the-rule corneal astigmatism which did not change following surgery. Kwitko *et al. *[16] have reported steepening of inferior and inferotemporal cornea with flattening of superior cornea following inferior rectus recession in Grave’s disease. Progressive myopia has been reported in 2 patients of TO by Huismans [8]. The author suggested edema and infiltration of lymphocytes of and plasmacytes of ciliary-body, pathologic-anatomical substratum of the most changes of orbita in this disease, responsible for myopia. Chandrasekaran *et al. *[5] have reported 5 cases of acquired hypermetropia in TO which reversed following orbital decompression. They proposed that the acquired hypermetropia was due to the increased volume of orbital contents with flattening of posterior globe.

Proptosis displaces the orbital contents outside the bony orbit producing a decompressive effect. Significant Proptosis may therefore reduce congestion and help reduce IOP in comparison to “tight orbits”. Changes in IOP appear to be more pronounced in patients with congestive ocular symptoms and this, in addition to calculation of orbital volume may predict the outcomes of orbital decompression with respect to IOP and refraction. As suggested by Robert *et al. *[6] a prospective study including orbital volume calculations using 3D imaging studies would provide more accurate information on the indications for orbital decompression procedures.

Our study demonstrated a significant reduction in proptosis following orbital decompression consistent with previous reports [6,17-20], however we did not find a significant change in IOP and refraction following orbital decompression for TO. The limitations of our study were its retrospective design, lack of IOP data in all 3 positions of gaze, lack of corneal thickness measurements and the small number of patients. Taking into account the shortcomings of our study we have proposed a prospective study including IOP measurements performed with tonopen at the same time of day in 3 different positions of gaze using fellow eye as a control. Our study did not find a significant change in refraction following orbital decompression for thyroid orbitopathy, either. However, this study also helped identify inadequacy in all retrospective decompression-IOP studies published so far, including the need to record corneal pachymetry, diurnal variation, ensure central corneal IOP measurements are only taken and standardise measurements in 3 positions of gaze, primary, up and down gaze. A prospective study is therefore required to evaluate IOP change.

## Figures and Tables

**Table 1:** Reduction in Proptosis and Change in Intraocular Pressure (IOP) in Relation to the Type of Orbital Decompression Procedure Carried Out

Decompression Procedure	Mean Volume of Fat Excised (ml)	Mean Reduction in Proptosis (mm)	Mean Preoperative IOP (mmHg)	Mean IOP at 1 Week After Surgery (mmHg)	Mean IOP at 3 Months After Surgery (mmHg)
Intraconal fat excision only (6 eyes)	2.0 ± 0.36	2 ±0.8	14.5±0.5	15±1.4	15.5±1.9
2 wall bone + fat excision (7 eyes)	1.5 ± 0.8	4 ± 1.1	18.2±2.8	18.6±1.0	17.1±2.7
3 wall bone + fat excision (5 eyes)	1.6 ± 0.5	5 ± 2.6	17.4±3.7	17.5±3.7	17.4±3.2

**Table 2:** Comparison of Pre and Post Operative Autorefraction and Keratometry

	AR 1	AR 2	Axis	K1	K2	Axis
Median (range) Preoperative	-0.25 D (-1.75 to 2.75)	-0.5 D (-3 to -0.5)	47.5 (4 to 160)	43.5 D (39.5-45.75)	44.5 D (41.5-47.25)	91.5 (8 to 165)
Median (range) Postoperative	0.0 D (-2.25 to 3)	-0.75 D (-3.75 to 0.5)	62.5 (1 to 180)	44 D (40 to 45.25)	44.75 D (42.5 to 47.25)	90.5 (7 to 135)
P (Wilcoxon Signed Rank Test)	p = 0.89	p = 0.76	p = 0.42	p = 0.20	p = 0.06	p = 0.45

Values following orbital decompression.

AR= autorefraction; K= keratometry.
